# Reactive oxygen species generation in aqueous solutions containing GdVO_4_:Eu^3+^ nanoparticles and their complexes with methylene blue

**DOI:** 10.1186/s11671-018-2514-5

**Published:** 2018-04-13

**Authors:** Kateryna Hubenko, Svetlana Yefimova, Tatyana Tkacheva, Pavel Maksimchuk, Igor Borovoy, Vladimir Klochkov, Nataliya Kavok, Oleksander Opolonin, Yuri Malyukin

**Affiliations:** Institute for Scintillation Materials National Academy of Sciences of Ukraine, 60 Nauky ave., Kharkiv, 61072 Ukraine

**Keywords:** Reactive oxygen species, Nanoparticles, Photocatalytic activity, Radical scavenging

## Abstract

It this letter, we report the study of free radicals and reactive oxygen species (ROS) generation in water solutions containing gadolinium orthovanadate GdVO_4_:Eu^3+^ nanoparticles (VNPs) and their complexes with methylene blue (MB) photosensitizer. The catalytic activity was studied under UV-Vis and X-ray irradiation by three methods (conjugated dienes test, OH· radical, and singlet oxygen detection). It has been shown that the VNPs–MB complexes reveal high efficiency of ROS generation under UV-Vis irradiation associated with both high efficiency of OH· radicals generation by VNPs and singlet oxygen generation by MB due to nonradiative excitation energy transfer from VNPs to MB molecules. Contrary to that under X-ray irradiation, the strong OH^**.**^ radicals scavenging by VNPs has been observed.

**Electronic supplementary material:**

The online version of this article (10.1186/s11671-018-2514-5) contains supplementary material, which is available to authorized users.

## Background

Radiation therapy (RT) remains an important component of cancer treatment with approximately 50% of all cancer patients receiving RT during their course of illness [1–3]. The exact mechanism of cell death due to radiation is still an area of active investigation. Double-stranded breaks of nuclear DNA are considered to be the most important cellular effect of radiation leading to irreversible loss of the reproductive integrity of the cell and eventual cell death [4]. Such radiation damage can be caused by (i) direct ionization and (ii) indirect ionization via free radicals and reactive oxygen species (ROS), chemically reactive species containing oxygen, formed from the radiolysis of cellular water and oxygen molecules [2–4]. In clinical therapy, damage is commonly indirect ionizing. In the process, water loses an electron and becomes highly reactive. Then, through a three-step chain reaction, water is sequentially converted into a number of radicals and molecular products: hydrated electrons (${e}_{aq}^{-}\Big)$, hydrogen atom( *H*^∙^), hydroxyl radical OH·, hydroperoxyl radical (${HO}_2^{.}\Big)$, hydrogen peroxide (H_2_O_2_), and hydrogen molecules (H_2_) [5, 6]. Hydrated electrons and hydrogen atoms are strong reducing agents. In contrast, hydroxyl radicals are very strong oxidative species and immediately remove electrons from any molecule in its path, turning that molecule into a free radical and thus propagating a chain reaction [5]. When dissolved molecular oxygen is presented in irradiated water, its reduction produces superoxide radical (${O}_2^{.-}$) and is the precursor of most other ROS including singlet oxygen (^1^O_2_) [7].

Recently, it has been shown that high atomic number (Au, Ag, Hf, Gd, Ti based) nanoparticles (NPs) [8–11], semiconductor NPs (metal-oxide TiO_2_ ZnO, CuO, CeO_2_, Al_2_O_3_; quantum dots ZnS, ZnS, LaF_3_, etc.) [8, 12–14], and some inorganic NPs (carbon nanotubes) [15, 16] enhance the efficiency of RT. Theoretical principles of X-rays–NP interaction are well described [8, 12, 14]. A cascade interaction of high-energy photons with the NP’s lattice occurs through the photoelectric effect and the Compton scattering effect mainly. Compton, Photo-, or Auger-electrons can induce the emission of secondary electrons, which can escape into the environment and will be captured by an acceptor (i.e., water, biomolecule, oxygen, nitrogen oxides) localized near the surface of NPs and induce biomolecular radicals and ROS production [8, 12, 14]. Radiosensitizing effects of NPs is associated with biomolecular radicals and ROS generation as a final stage of X-ray interaction with NPs. In semiconductor NPs, such as metal-oxide NPs, the cytotoxic effect associated with ROS generation can be also induced by UV irradiation [17–20]. The mechanism is that when NPs are irradiated with the UV light (energy greater than the band gap), the charge separation is induced generating a hole (h^+^) in the valance band and an electron (e^−^) in the conducting band. Electrons and holes exhibit high reducing and oxidizing ability, respectively [18]. The electrons can react with molecular oxygen to produce superoxide radical (${O}_2^{\cdotp -}$) through a reductive process, whereas the holes can abstract electrons from water and/or hydroxyl ions generating hydroxyl radicals (OH·) through an oxidative process [18–20]. For TiO_2_, CeO_2_, Al_2_O_3_, and ZnO nanoparticles, the ^1^O_2_ generation via the oxidation of ${O}_2^{\cdotp -}$ was reported [18, 21, 22].

One more approach to enhance the efficiency of cancer therapy (photodynamic therapy, PDT) using scintillating NPs was proposed by Chen and Zhang [23]. This approach combines X-ray excited (scintillating) NPs and photosensitizer (PS) molecules. Scintillating NPs serve as an energy transducer transferring energy harvested from X-ray irradiation to the PS that generates singlet oxygen for tumor destruction. This approach allows deep cancer treatment and enhances both PDT and RT. Up to now, a variety of scintillation NPs and their complexes with PS have been studied as X-ray inducible photodynamic agents [12, 14, 23–28].

Recently, we have reported on the creation of complexes on the base of scintillating gadolinium orthovanadate NPs doped with europium ions GdVO_4_:Eu^3+^ (VNPs) and methylene blue (MB) photosensitizer and study of electronic excitation energy transfer in the complexes [29]. It was shown that due to the effective excitation energy conversion in the complexes, they could be prospective as an X-ray inducible photodynamic agent. The aim of this paper was to study the efficiency of ROS generation in water solutions containing VNPs and their complexes with MB under UV and X-ray irradiation.

## Experimental

### Chemicals

Gadolinium chloride GdCl_3_·6H_2_O (99.9%), europium chloride EuCl_3_·6H_2_O (99.9%), disodium EDTA·2Na (99.8%), and anhydrous sodium metavanadate NaVO_3_ (96%) were obtained from Acros organic (USA) and all used without further purification. Sodium hydroxide NaOH (99%) was purchased from Macrohim (Ukraine). Sodium orthovanadate Na_3_VO_4_ solution was obtained by adding a 1 M solution of NaOH in aqueous solution NaVO_3_ to pH = 13. L-a-phosphatidylcholine (PC) from egg yolk, cationic dye 3,7-bis(dimethylamino)phenazathionium chloride (methylene blue (MB), M_w_ = 373.90 g/mol), 1,2-Benzopyrone (Coumarin, M_w_ = 146.14 g/mol) were purchased from Sigma-Aldrich (USA) and used as received. Antracene-9,10-dipropionic acid disodium salt (ADPA, M_w_ = 366.32 g/mol) was obtained from the dye collection of Dr. Igor Borovoy (Institute for Scintillation Materials, NAS of Ukraine) with the purity controlled by thin layer chromatography. All other chemicals were of analytical grade.

### Synthesis of GdVO_4_:Eu^3+^ colloidal solutions

Aqueous colloidal solutions of gadolinium orthovanadate nanoparticles doped with europium ions Gd_0.9_Eu_0.1_VO_4_ (GdVO_4_:Eu^3+^) were synthesized according to the method reported earlier [30]. First, 0.4 mL of aqueous solution of gadolinium chloride (1 M) was mixed with 0.05 mL of europium chloride (1 M) then 49.55 mL of doubly distilled water was added to the mixture. Then, obtained solution was mixed with 37.5 mL of disodium EDTA solution (0.01 M). Then, 37.5 mL of Na_3_VO_4_ (0.01 M) was flowed drop wise (рН = 10.5). The mixture was intensively stirred by using a magnetic stirrer and heated on a water bath under a reflux condenser for 24 h at 100 °С. Obtained colorless transparent solution scatters light under the side illumination (the Tyndall cone). Then, the solution was cooled and dialyzed against water for 24 h to remove the excess of ions. For this purpose, the obtained solution was purred in a dialysis sac (Cellu Sep T2, membrane with a molecular weight cut-off of 12 KDa, pore size ~ 2.5 nm) and placed in a 2 L glass with distilled water. After each 6 h, the water was refreshed.

### Instrumentation and characterization

Synthesized VNPs were characterized using transmission electron microscopy (TEM-125 K electron microscope, Selmi, Ukraine) and dynamic light scattering method (ZetaPALS analyzer, Brookhaven Instruments Corp., USA). Absorption spectra were measured using a Specord 200 spectrometer (Analytik Jena, USA). Fluorescence and fluorescence excitation spectra were taken with a spectrofluorimeter Lumina (Thermo Scientific, USA).

### Preparation of VNPs–MB complexes

Solutions for investigations were prepared as follows. First, stock solutions of MB in water (1 mmol/L) were prepared. To obtain VNPs–MB aqueous solutions, required amount of the dye stock solution and VNPs aqueous solution were added in a flask and carefully stirred using a rotary evaporator (Rotavapor R-3, Buchi) during 1 h to a complete evaporation of chloroform. Then, 1 mL of a VNPs aqueous solution was added in a flask and gently shaken during 1 h for VNPs–MB complex formation. The concentration of MB in the obtained solution was 10 μmol/L. The concentrations of nanoparticles were 0.1, 1, or 10 mg/mL.

### Active oxygen and free radical species detection

The formation of ROS under the UV/X-ray irradiation of aqueous solutions containing VNPs, MB, or VNPs-MB complexes was detected spectroscopically using several methods.

#### Conjugated dienes formation test

Lipid oxidation under UV irradiation was measured using PC liposomes suspension. Unilamellar PC lipid vesicles were prepared by the extrusion method [31]. Briefly, appropriate amount of PC (25 mg/ml) in chloroform was placed in a flask and dried until complete chloroform evaporation using a rotary evaporator (Rotavapor R-3, Buchi). The thin lipid-dyes film was then hydrated with 10.8 ml of double distilled water. The obtained lipid suspension was finally extruded through 100 nm pore size polycarbonate filter using a mini-extruder (Avanti Polar Lipids, Inc., USA). The concentration of PC was 1.2 mmol/L. For conjugated dienes formation test, 1 mL of the PC liposome suspension was mixed with 1 mL of VNPs water solution (MB water solution or VNPs–MB water solution). The final MB concentration was 10 μmol/L and VNPs, 1 g/L. PC concentration in the solutions was 0.6 mmol/L. The obtained aqueous solutions were placed in quartz cuvettes (10 × 10 mm) and irradiated with 250 W mercury lamp (band pass *l* = 310–400 nm, light flux was 43 W/cm^2^) for 30 min. Then, the absorbance of the suspensions was recorded at 234 nm (conjugated dienes maximum) using a Specord 200 spectrophotometer (Analytik Jena, Germany). The concentration of conjugated dienes formed in water without any additives (NPs, MB, or VNPs–MB complexes) was taken as a control. Each experimental point was the mean value of at least three independent tests. Statistical processing was carried out using the software package Statistika v. 5.0 (StatSoft, USA).

#### OH· radical detection

To detect the hydroxyl radical formation in the solution under UV irradiation, coumarin was used as a probe molecule. Coumarin reacts with OH· radicals producing highly fluorescent 7-hydroxycoumarin [32, 33]. Experimental procedure was as follows. Coumarin aqueous solution (0.1 mmol/L) was mixed with MB (10 μmol/L), VNPs (0.1, 1 or 10 g/L), or VNPs–MB aqueous solutions. The obtained aqueous solutions were placed in quartz cuvettes (10 × 10 mm) and irradiated with He-Cd laser *λ*_exc_ = 325 nm for 1 h. In case of X-ray irradiation, the cuvette was irradiated from above (from the open part) by an X-ray using ISOVOLT 160 Titan E apparatus with a tungsten cathode for 30 min. The voltage on the tube was 30 kV (20 mA). The distance from the X-ray tube to the irradiated samples was 25 cm. The fluorescence spectra (excited at 325 nm) of the solutions were recorded with a spectrofluorimeter Lumina (Thermo Scientific, USA). The relative intensity of 7-hydroxycoumarin fluorescence was analyzed.

#### Singlet oxygen detection

^1^O_2_ production in the solutions containing VNPs, MB, or VNPs–MB complexes was analyzed on the evaluation of ADPA fluorescence spectra [34, 35]. The measurements were carried out in quartz cuvettes (10 × 10 mm). ADPA aqueous solution (10 μmol/L) was mixed with MB (10 μmol/L), NPs (1 g/L), or VNPs–MB aqueous solutions in a cuvettes. The solutions were irradiated at 457 nm using High Stability Blue Solid State Laser MBL-457, 50 mW (Changchun New Industries Optoelectronics Tech. Co., Ltd.). The fluorescence emission of ADPA excited at 378 nm was collected at different time scales (0, 10, 20, 30, 40, and 60 min) using a spectrofluorimeter Lumina (Thermo Scientific, USA).

## Results and discussions

### Characteristic of synthesized VNPs

**Fig. 1:**
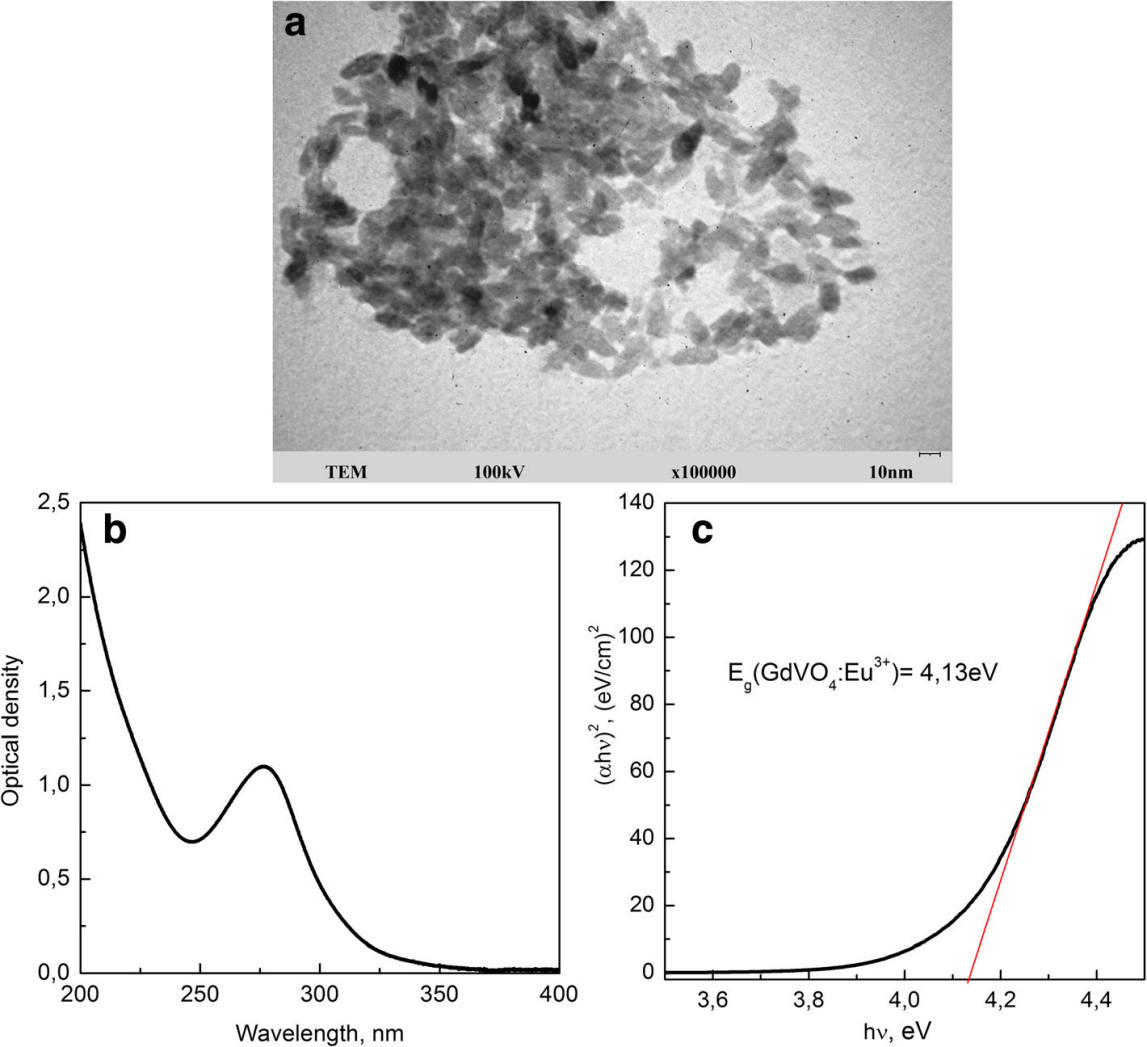
TEM image (**a**), absorption spectrum (**b**), and energy dependence of (*αhv*)^2^ (**c**) of GdVO_4_:Eu^3+^ nanoparticles

Figure 1 a and Additional file 1: Figure S1 show the TEM images of synthesized VNPs with a side distribution histogram and an XRD pattern, which support the GdVO_4_:Eu^3+^ NPs crystalline structure. Synthesized GdVO_4_:Eu^3+^ NPs are of spindle-like form with a 8 × 25 nm ± 5 nm size and tetragonal phase structure of zircon type. The negative charge of the NPs surface (ζ-potential is − 18.75 ± 0.15 mV, pH = 7.8) is due to carboxylate groups of disodium EDTA stabilizer used during the synthesis. The overage hydrodynamic diameter of GdVO_4_:Eu^3+^ nanoparticles is 44.0 ± 0.3 nm. The absorption spectrum of GdVO_4_:Eu^3+^ NPs represents of the intense wide band in the 250–350 nm spectral range that corresponds to a charge transfer from oxygen ligands to the vanadium atom in ${VO}_4^{3-}$ group (Fig. 1b) [36]. Doping GdVO_4_ NPs with Eu^3+^ ions imparts strong fluorescence to VNPs in the red spectral range, which is governed by the transition within the f–electron configuration of the europium ions [37] (will not be discussed in this paper).

It is known that the size of NPs affects the optical energy gap in semiconductor materials. The band gap energy, *E*_*g*_, can be estimated from the absorption edge wavelength of the inter-band transition according to the Tauc’s relationship [38]:

1 $$ {\left(a\mathrm{hv}\right)}^{\left(1/n\right)}=A\cdot \left(\mathrm{hv}-{E}_g\right), $$

where a is absorption coefficient, hv is the incident photon energy, *A* is the energy independent constant (the band tailing parameter), and *n* is the constant (power factor of the transition mode), which depends on the material nature (crystalline or amorphous). The value of *n* denotes the nature of the transition, *n* = 1/2 for direct allowed transitions, *n* = 3/2 for direct forbidden transitions, *n* = 2 for indirect allowed transitions, and *n* = 3 for indirect forbidden transition [39]. GdVO_4_ is a direct gap semiconductor, for which *n* = 1/2 [40] Thus, Eq. (1) can be rewritten as:

2 $$ {\left(\alpha \mathrm{hv}\right)}^2=A\cdot \left(\mathrm{hv}-{E}_g\right) $$

Absorption coefficient (*a*) is calculated from absorbance as *a* = 2.303*D*/*l*, where *D* is absorbance and *l* is the optical pathlength.

Figure 1c represents the energy dependence of (*a*hv)^2^ for synthesized GdVO_4_:Eu^3+^ nanoparticles. The band gap value *E*_*g*_ was determined by extrapolation of the linear portion of the (*a*hv)^2^ curve versus the photon energy hv to zero. The obtained value *E*_*g*_ = 4.13 *eV* is higher than that reported for GdVO_4_:Eu^3+^ powders with crystallite size ranging from 14.4 to 43 nm (3.56–3.72 eV) [41, 42]. We suppose it could be due to the difference in used synthesize methods that in our case gives smaller NPs with narrow and blue-shifted absorption band as compared to that obtained by the hydrothermal or Pechini’s methods.

### Photo-induced free radicals generation (conjugated dienes test)

It is commonly accepted that tree types of ROS ($\mathrm{OH}\cdotp, {\mathrm{O}}_2^{\cdotp -}$, and ^1^O_2_ ) generating in NPs systems under UV irradiation contribute to the major oxidative stress in biological systems [43, 44]. Although photocatalytic activity of such metal-oxide NPs as TiO_2_, ZnO, CuO, CeO_2_, Al_2_O_3_, and Fe_2_O_3_ is well-described [17–20], little research has studied the photocatalytic activity of ReVO_4_ NPs [45–48]. It was shown that ReVO_4_ NPs are effective in photocatalytic destruction of organic pollutants. However, no research has studied the types of ROS generated by ReVO_4_ NPs under UV irradiation.

**Fig. 2:**
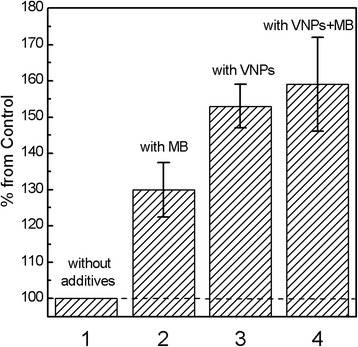
Relative efficiency of conjugated dienes formation in lipid suspensions: 1 - without additives; 2 - with MB; 3 - with VNPs; 4 - with VNPs-MB complexes

To mimic biological environment, we used PC liposome suspension and detected free radicals generation under UV irradiation in the suspensions containing MB, VNPs, or VNPs–MB complexes on lipid oxidation (conjugated dienes formation test) [49–51]. Lipid oxidation by molecular oxygen via radical chain reactions can be initiated by ionizing radiation when ROS and free radicals appear in the system [43, 44]. Radical chain reactions involving polyunsaturated fatty acids cause a rearrangement of the double bonds leading to conjugated dienes. The resulting conjugated dienes exhibit an absorption band at 234 nm that could be detected photometrically. Figure 2 shows relative concentrations of conjugated dienes formed in lipid suspensions containing MB, VNPs, or VNPs–MB complexes. It could be seen that in all solutions, the concentration of conjugated dienes increases as compared to the pure PC liposome suspension. However, the efficiency of this process differs. Methylene blue is one of the conventional photosensitizer molecules with the main absorption maxima *λ*_max_ = 665 nm and a less intense absorption band in the UV spectral range (Additional file 1: Figure S2). Under UV irradiation of MB, the two major photochemical processes may take place [34, 52]. MB excited by UV light undergoes intersystem crossing process (*Q*_*p*_=0.54 [53]) to the long-lived triplet state (^3^MB^*^) and reacts with oxygen molecules (^3^O_2_) forming singlet oxygen (^1^O_2_):

3 $$ {\mathrm{MB}}^{+}+ hv\to {}^3{\mathrm{MB}}^{+^{\ast }} $$

4 $$ {}^3{\mathrm{MB}}^{+^{\ast }}+{}^3{\mathrm{O}}_2\to {\mathrm{MB}}^{+}+{}^1{\mathrm{O}}_2 $$

The second photochemical process may take place at high MB concentrations. The ground state MB molecules can work as reducing agents donating an electron to the MB triplet and forming the semi-reduced radical (MB·) and semi-reduced radical, respectively (MB^**·**2+^) [52]:

5 $$ {}^3{\mathrm{MB}}^{+^{\ast }}+{\mathrm{MB}}^{+}\to \mathrm{MB}\cdotp +{\mathrm{MB}}^{\cdotp 2+} $$

The oxidation of MB· by molecular oxygen returning the ground state dye and leading to superoxide radical production (${O}_2^{\cdotp -}$):

6 $$ \mathrm{MB}\cdotp +^3{\mathrm{O}}_2\kern0.5em \to {\mathrm{MB}}^{+}+{O}_2^{\cdotp -} $$

Singlet oxygen and superoxide radicals, as well as MB radicals formed in Reactions (4)–(6) can affect the lipid oxidation process. In diluted solution where no MB dimer formation is observed ([MB] < 20 μM), Reactions (3) and (4) will dominate [52]. However, in VNPs–MB complexes due to increased MB concentration within VNPs surface [29], the second photochemical process can take place. Thus, the increase of the conjugated diene formation in the lipid suspension containing MB can be explained by MB action as ^1^O_2_ photogenerator under UV irradiation. It should be noted that the efficiency of this process is much smaller than that under MB excitation within long-wavelength absorption maximum.

In the suspension containing GdVO_4_:Eu^3+^ nanoparticles, lipid oxidation is more effective. This effect could be ascribed to the photocatalytic behavior of VNPs under UV irradiation. Conducting band electrons (e^−^) and valence band holes (h^+^) formed under UV irradiation (*E* > *E*_*g*_) can interact with molecular oxygen and water molecules adsorbed on the NPs surface by following reactions [18, 20, 47]:

7 $$ {}^3{\mathrm{O}}_2+{e}^{-}\to \kern0.5em {\mathrm{O}}_2^{.-} $$

8 $$ {\mathrm{H}}_2\mathrm{O}+{\mathrm{h}}^{+}\to \mathrm{OH}\cdot $$

Hydroxyl ions formed during water photolysis and adsorbed on NPs surface can also interact with holes to produce hydroxyl radicals:

9 $$ {\mathrm{OH}}^{-}+{\mathrm{h}}^{+}\to \mathrm{OH}\cdot $$

Moreover, the oxidation of ${O}_2^{\cdotp -}$ produces singlet oxygen [18, 21, 22]:

10 $$ {\mathrm{O}}_2^{\cdotp -}+{\mathrm{h}}^{+}{\to}^1{\mathrm{O}}_2 $$

Its reaction with hydrogen ions leads to hydrogen peroxide formation:

11 $$ 2{\mathrm{O}}_2^{\cdotp -}+2{\mathrm{H}}^{+}\to {\mathrm{H}}_2{\mathrm{O}}_2 $$

as a result of its interaction with electrons hydroxyl radicals and hydroxyl ions can be formed:

12 $$ {\mathrm{H}}_2{\mathrm{O}}_2+{\mathrm{e}}^{-}\to \mathrm{OH}\cdot +{\mathrm{O}\mathrm{H}}^{-} $$

Thus, the increase in efficiency of conjugated dienes concentration in a suspension containing VNPs (Fig. 2, column 3) can be ascribed to the products generating via Reactions (7)–(12) and facilitating lipid oxidation.

In the lipid suspension containing complexes VNPs–MB, the highest conjugated dienes concentrations can be explained by products generated both via Reactions (3)–(6) and Reaction (7)–(12) (Fig. 2, column 4). Moreover, in VNPs–MB complexes in Reaction (3) and (4), singlet oxygen generation could take place both due to direct MB excitation and via nonradiative excitation energy transfer from VNPs to MB that is rather effective in this composition [29].

### Hydroxyl radical detection

**Fig. 3:**
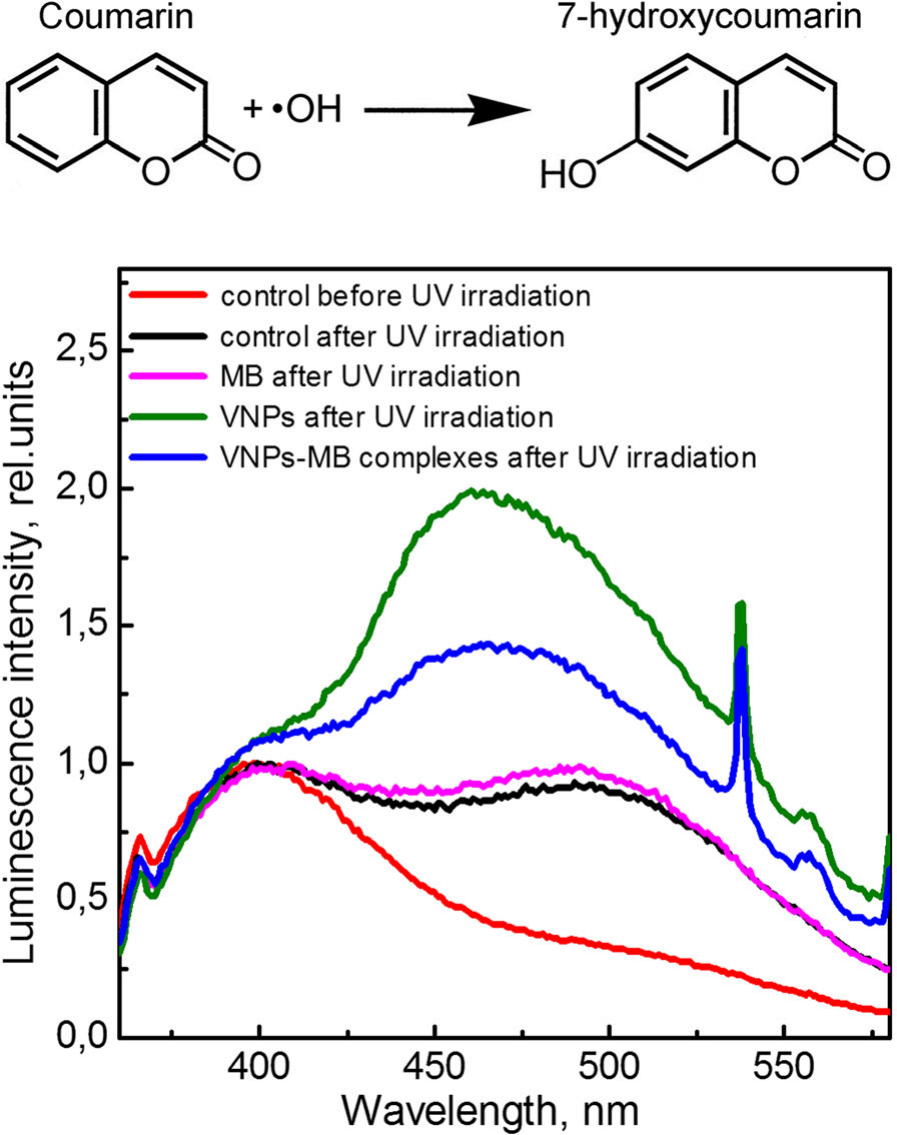
Reaction of coumarin with hydroxyl radical to form fluorescent 7-hydroxycoumarin. Normalized fluorescence spectra of coumarin water solution, *λ*_exc_ = 325 nm

The next step was to examine more exactly the efficiency of OH· and ^1^O_2_ generation in the solutions under UV/X-ray irradiation. Coumarin was used as a probe molecule to validate the appearance of hydroxyl radicals in the solutions under consideration. It is known that OH· radicals are one of the main products of water photolysis/radiolysis under UV/X-ray irradiation [5, 6]. In water solution, OH· radicals interact with coumarin molecules to form highly fluorescent product 7-hydroxycoumarin (see scheme in Fig. 3) that could be detected spectroscopically by the appearance of a new band (*λ*_max_ ~ 460 nm) shifted toward the long-wavelength spectral region with respect to the coumarin fluorescence band (*λ*_max_ ~ 400 nm), Fig. 3 [32, 33]. The higher the concentration of OH· radicals in the solution is, the more effective coumarin oxidation and, consequently, the more intense the long-wavelength band are. Thus, analysis of the relative intensity of the long-wavelength band could provide the information about the concentration of OH· radicals in the solution under effect of various factors.

The fluorescence emission spectra of the coumarin water solution containing MB, VNPs, or VNPs–MB complexes measured after 1 h of UV illumination is presented in Fig. 3. It is shown that UV irradiation of coumarin water solution without any additives (control) provokes a formation of a new long-wavelength fluorescence band that indicates OH· radicals generation and coumarin oxidation (Fig. 3). In the presence of MB molecules in the solution, the relative intensity of this band does not change that indicates no additional effects of MB on OH· radicals generation (Fig. 3). In the solution containing VNPs, the intensity of the 7-hydroxycoumarin band increases remarkably (Fig. 3) due to photocatalytic activity of VNPs under UV irradiation, Reactions (8), (9) and (12). Let us note that the sharp peaks around 535–540 nm belong to the europium ion fluorescence in GdVO_4_:Eu^3+^ nanoparticles (intraconfiguration transitions). In the solution containing VNPs–MB complexes, the relative intensity of the 7-hydroxycoumarin band was about twice as smaller as compared to that in the solution containing VNPs that points to the less effective OH· radicals production (Fig. 3). That can be explained by the fact that the MB dye adsorption within the VNPs surface can prevent water molecules and hydroxyl ions adsorption and, consequently, reduces VNPs photocatalitic activity concerning OH· radicals generation via Reactions (8) and (9). Moreover, in VNPs–MB complexes, a part of adsorbed energy is transferred nonradiatively to MB molecules [29] that also decreases the efficiency of electron-hole pairs production and, consequently, VNPs capability for OH· radicals generation in such complexes.

**Fig. 4:**
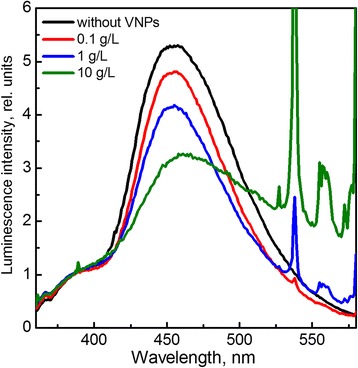
Normalized fluorescence spectra of coumarin water solution containing different concentration of VNP recorded after 30 min X-ray irradiation

Unexpected results were observed under X-ray irradiation of the solutions containing VNPs (Fig. 4). Contrary to the case of UV irradiation, we observe that the relative intensity of 7-hydroxycoumarin band decreases as compared to the coumarin water solution without nanoparticles that indicates the scavenging of OH· radicals formed in the solutions as a result of water radiolysis. The observed effect is strongly depended on the VNP concentrations (Fig. 4). It should be noted that the main discussion concerning nanoparticles’ ability to serve as a ROS scavenger is focused mainly on CeO_2_ nanocrystals (nanoceria) [54–57]. The main features that forces nanoceria to act as ROS scavenger are generally attributed to high content of oxygen vacancies and Ce^3+^ ions in nanoceria and its switching between 3+ and 4+ oxidation states. However, the critical dependence of nanoceria biological activity on its size and self-regeneration mechanism is still under discussion [54–57]. We note also that protective effects of GdVO_4_:Eu^3+^ and CeO_2_ NPs against X-ray-induced damages were observed in our group earlier in vivo experiments [57]. To the best of our knowledge, the ability of GdVO_4_:Eu^3+^ nanoparticle to sweep OH· radicals generated in the water solution under X-ray irradiation has been observed for the first time and requires further more in-depth research.

### Singlet oxygen generation

**Fig. 5:**
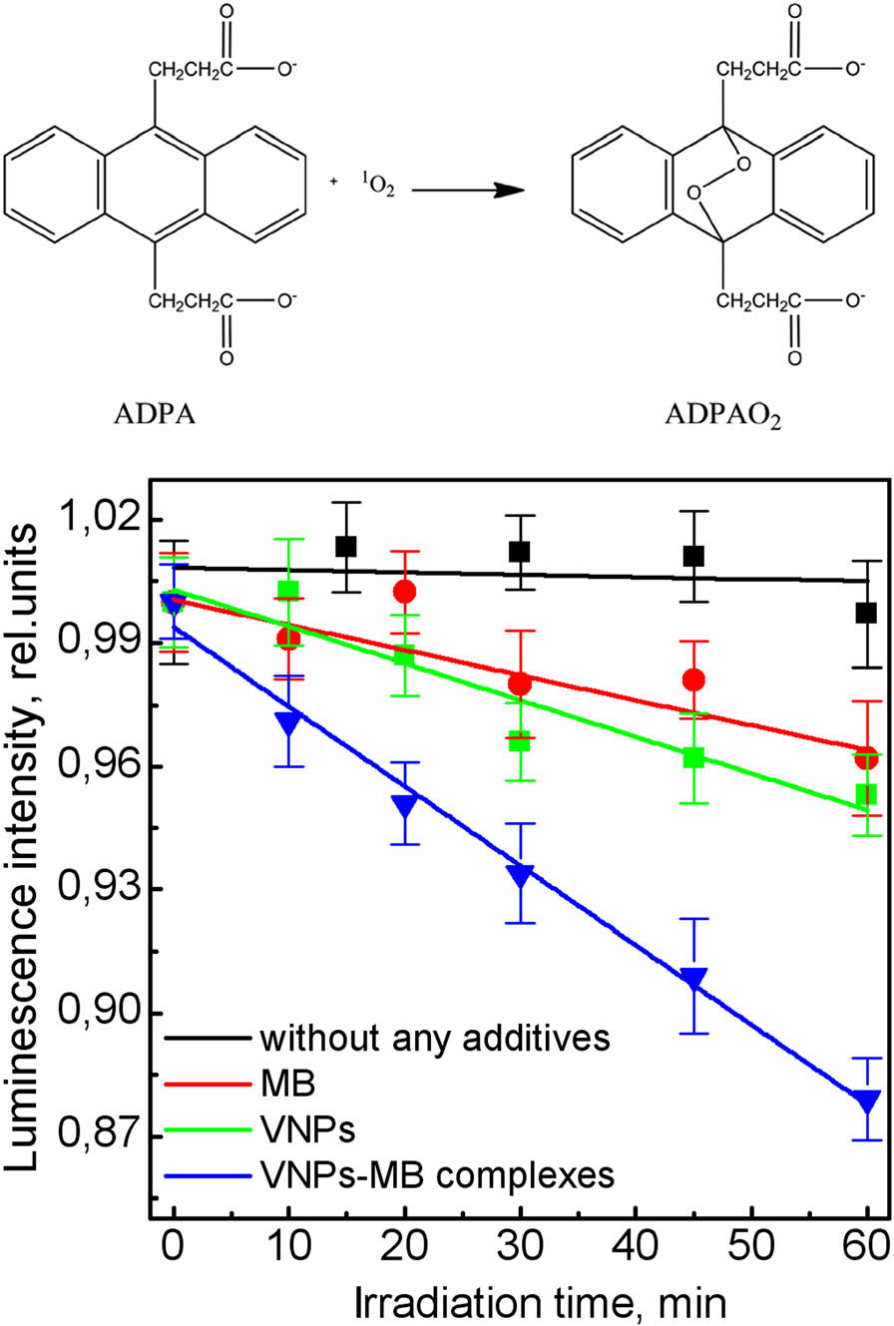
Reaction of ADPA with singlet oxygen to form endoperoxide ADPAO_2_. ADPA photobleaching after irradiation with *λ* = 457 nm in water solutions

To evaluate the efficiency of VNPs–MB complexes of ^1^O_2_ generation in water, we use the method-based ADPA oxidation by singlet oxygen with a formation of non-fluorescent endoperoxide ADPAO_2_ (Fig. 5). Thus, in the presence of singlet oxygen, the ADPA fluorescence is quenched irreversibly. We should note that under UV irradiation, ADPA molecules undergo strong photobleaching that complicates the identification of MB, VNPs, or VNPs–MB complexes impacts associated with the ^1^O_2_ generation. To overcome this drawback, we apply laser irradiation at 457 nm, which matches one of the excitation peaks of Eu^3+^ ions doped in GdVO_4_ nanocrystals (Additional file 1: Figure S3). Figure 5 shows that, the ADPA molecules undergo no photochemical reactions at the irradiation of 457 nm light. In the solution containing MB, a slight decrease of ADPA intensity in time could be observed (Fig. 5) that is associated with MB slight excitation at this wavelength and action as photosensitizer according to Reaction (3) and (4). The same effect is observed for the solution containing VNPs (Fig. 5) and could be ascribed to the formation of ${O}_2^{\cdotp -}$ radicals on the surface of VNPs (Reaction (7)) followed by its oxidation according to Reaction (10) with singlet oxygen generation. The stronger ADPA fluorescence quenching is observed in VNPs–MB complexes. The efficiency of this process is twice as higher as in the solution with MB or VNPs. The higher efficiency of singlet oxygen generation in the solution containing VNPs–MB complexes is associated with the energy transfer from VNPs to the MB in the complexes, in which VNPs serve as energy transducer for MB photosensitizer.

Unfortunately, due to ADPA sensor instability, we were not successful to measure the efficiency of the ^1^O_2_ generation in water solution under X-ray excitation.

## Conclusions

The efficiency of ROS generation in water solutions containing GdVO_4_:Eu^3+^ nanoparticles and their complexes with MB have been analyzed under UV-Vis and X-ray irradiation by three methods (conjugated dienes test, OH· radical, and singlet oxygen detection). Complexes VNPs–MB reveal high efficiency of ROS generation under UV-Vis irradiation associated with both high efficiency of OH· radicals generation by VNPs and ^1^O_2_ generation by MB due to nonradiative excitation energy transfer from VNPs to MB molecules. For the first time, the strong OH· radicals scavenging by VNPs has been observed under X-ray irradiation. Our observation indicates that VNPs–MB complexes can be potentially used to activate photodynamic therapy.

## Additional file

Additional file 1: Supplementary materials. (DOCX 939 kb)

## Abbreviations

MB: Methylene blue
PS: Photosensitizer
ROS: Reactive oxygen species
VNPs: Gadolinium orthovanadate GdVO_4_:Eu^3+^ nanoparticles
