# Supplementary material for: Reactive oxygen species generation in aqueous solutions containing GdVO4:Eu3+ nanoparticles and their complexes with methylene blue
Source: Nanoscale Res Lett. 2018 Apr 13;13:100. doi: 10.1186/s11671-018-2514-5 (PMC5899080; doi:10.1186/s11671-018-2514-5)
Supplement: Supplementary file 1 — Supplementary materials. (DOCX 939 kb) [file 11671_2018_2514_MOESM1_ESM.docx]

**Reactive oxygen species generation in aqueous solutions containing GdVO_4_:Eu^3+^ nanoparticles and their complexes with Methylene Blue**

Kateryna Hubenko, Svetlana Yefimova^*^, Tatyana Tkacheva, Pavel Maksimchuk, Igor Borovoy, Vladimir Klochkov, Nataliya Kavok, Oleksander Opolonin, Yuri Malyukin

*Institute for Scintillation Materials National Academy of Sciences of Ukraine,*

*60 Nauky ave., 61072 Kharkiv,* *Ukraine*

**e-mail:* [*ephimova@isma.kharkov.ua*](mailto:ephimova@isma.kharkov.ua)


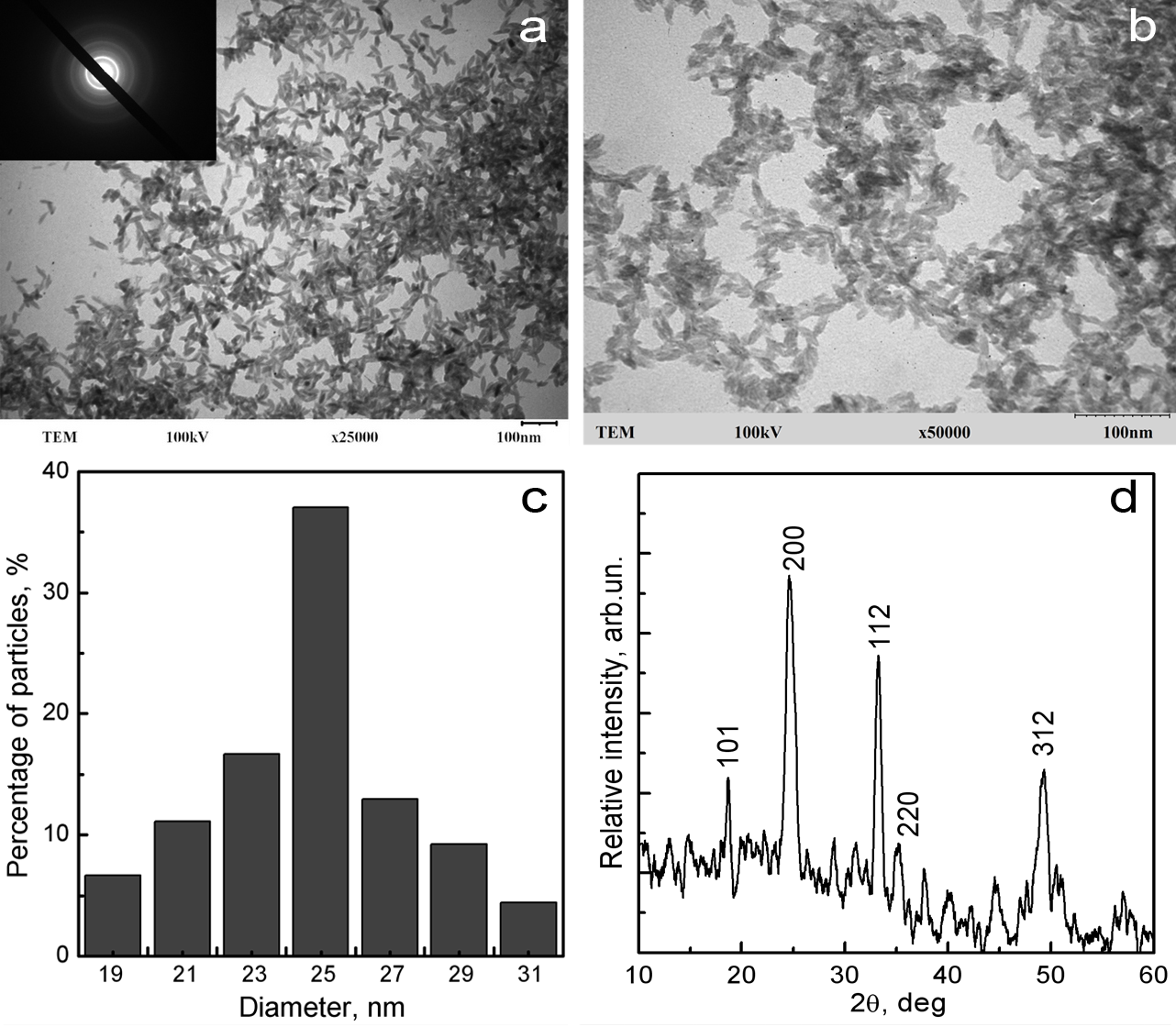


Fig. S1. TEM images of GdVO_4_:Eu^3+^ nanoparicles (a, b); histogram of size distribution (c); XRD pattern of GdVO_4_:Eu^3+^ nanoparticles (d).

Fig. S2. Absorption spectrum of MB.


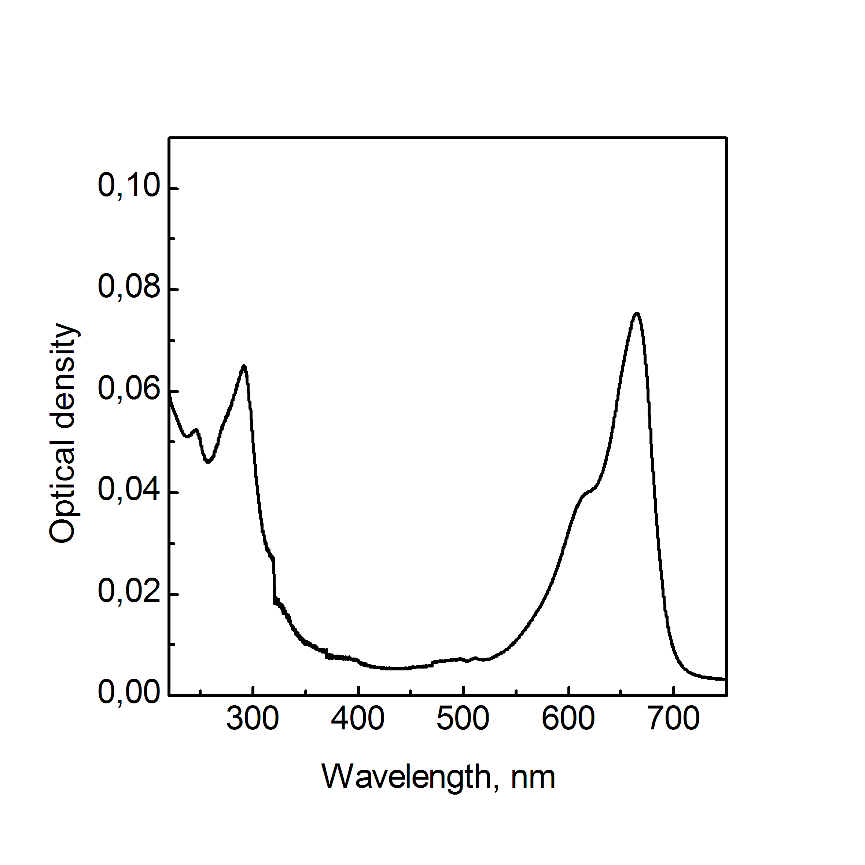

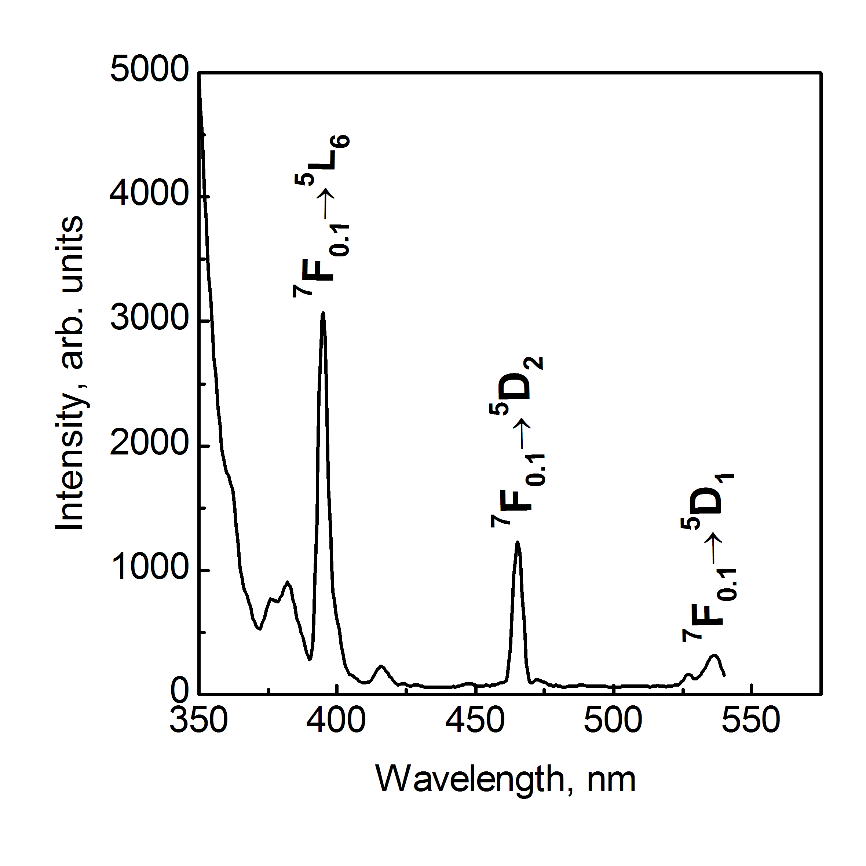


Fig. S3. Fluorescence excitation spectrum of GdVO_4_:Eu^3+^ nanoparticles.
